# Age modulates estradiol’s dual role in hepatocellular carcinoma recurrence after ablation: A prospective observational study

**DOI:** 10.1016/j.isci.2026.115823

**Published:** 2026-04-21

**Authors:** Xudong Gao, Jingfeng Bi, Shuwen Yang, Zhongyi Zhang, Na Liu, Wei Wu, Kun Yan, Jinghui Dong, Changchun Liu, Yun Zhu, Jiagang Huang, Zhen Zeng, MinHua Chen

**Affiliations:** 1 Department of Hepatology, the 5th Medical Center of Chinese PLA General Hospital, Beijing 100039, China; 2 Key Laboratory of Carcinogenesis and Translational Research (Ministry of Education), Department of Ultrasound, Peking University Cancer Hospital & Institute, Beijing 100142, China; 3 Department of Infectious Disease, The 5th Medical Center of PLA General Hospital, Beijing 100039, China; 4 The Fourth Retired Veteran Cadre’s Sanatorium, Beijing 100166, China; 5 Radiology Department, The 5th Medical Center of PLA General Hospital, Beijing 100039, China

**Keywords:** oncology, public health

## Abstract

The association between estrogen and hepatocellular carcinoma (HCC) remains controversial. This study explored estradiol (E2)’s effect on HCC recurrence post-minimally invasive treatment and age’s critical modifying role. 247 male HCC patients were prospectively enrolled, underwent radiofrequency ablation, and followed up for 36 months. Confounding factors were analyzed via cubic spline regression Cox model, with hierarchical analysis for age-specific effects. E2 and age showed a nonlinear correlation with HCC recurrence, with age identified as a confounding factor for E2. The cutoff values were 53/67 years (age) and 42 pg/ml (E2). For patients <53 years, E2 ≥42 pg/mL was linked to a significantly lower cumulative recurrence rate (CRR, *p* = 0.002); for 53–67 years (*p* = 0.065), no significant difference was observed; for >67 years, E2 ≥42 pg/mL was associated with a significantly higher CRR (*p* < 0.001). Thus, age modulates E2’s dual role: protective (<53 years), transitional (53–67 years), and risk-promoting (>67 years).

## Highlights

• By cubic spline Cox model analysis, age was identified as a confounding factor for E2
• Age modulates the bidirectional role of estrogen (E2) in tumor progression
• In the age-E2 relationship, cutoffs were 53/67 years and 42 pg/mL, respectively

## Graphical abstract

**Figure:**
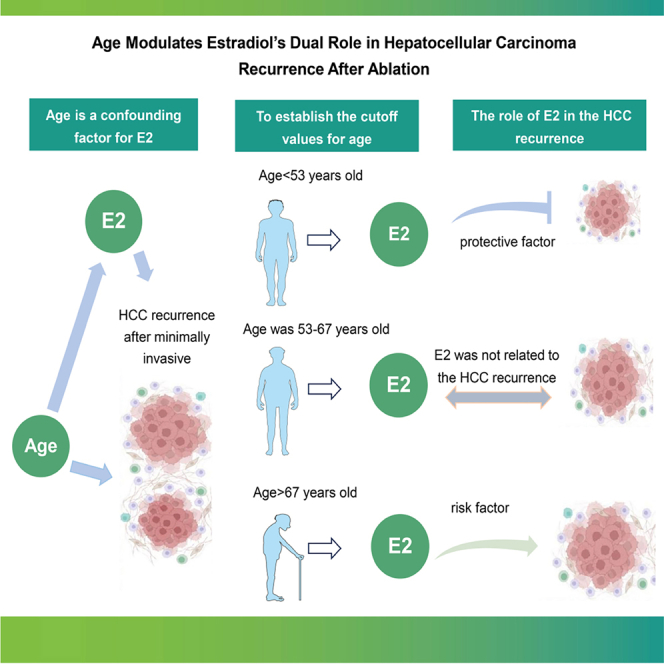

## Introduction

The role of sex hormones in cancer is an important issue that has been explored in many studies.^1^ Hepatocellular carcinoma (HCC) is closely related to sex hormones.^1^^,^^2^^,^^3^ Furthermore, sex has a direct impact on the occurrence of HCC^3^; most patients are male,^1^^,^^4^^,^^5^ suggesting that estrogen may play an important role in the occurrence and progression of HCC.

There are conflicting views on the relationship between estrogen and liver cancer.^1^^,^^3^ On the one hand, estrogen has been reported to have a protective effect.^6^^,^^7^ For example, epidemiological studies have found that the prognosis of female patients with HCC is significantly better than that of male patients with HCC,^3^^,^^8^ suggesting that estrogen may inhibit tumor progression.^8^ Further *in vitro* experiments have suggested that estradiol (E2) inhibits proliferation and increases apoptosis of Hep3B, BEL-7402, and Huh7 hepatoma cell lines^9^ and may inhibit the proliferation of hepatoma cells by inhibiting the expression of IL-6.^10^^,^^11^ 17β-E2 upregulates the NLRP3 inflammasome through the E2/ERβ/MAPK pathway, significantly inhibiting the tumor biological behavior of liver cancer cells.^12^^,^^13^ In addition, some studies suggest that E2 can inhibit the growth of liver cancer by regulating the polarization of macrophages.^14^^,^^15^ However, on the other hand, the contrasting view is that estrogen promotes the occurrence and progression of HCC. Long-term stimulation of exogenous estrogen can increase the incidence of HCC. Furthermore, estrogen leads to the activation of hepatic proto-oncogenes^16^ and is closely related to multiple miRNAs implicated in tumor progression.^17^^,^^18^ Some studies suggest that estrogen may activate the expression of mir-23a and p53 through ERα transcription,^17^ thus promoting tumor progression. The previous contradictory views have complicated the clinical understanding of the role of estrogen in patients with HCC, therefore hindering the clinical diagnosis and treatment of HCC. Hence, the role of estrogen in HCC needs to be further studied.

Considering that patients with liver cirrhosis exhibit impaired inactivation of estrogen, and some individuals present with hormone-related symptoms, such as menstrual disorders and sexual dysfunction, clinical hormone testing is routinely conducted. This study involved a long-term observation and follow-up of male patients with liver cancer scheduled to undergo radiofrequency ablation (RFA) treatment. We aimed to assess the impact of estrogen on tumor recurrence in individuals with HCC following radical minimally invasive treatment. This unique prospective observational study exclusively involved male patients. Due to the fluctuations in hormone levels, female patients were not included. Additionally, the study’s follow-up was limited to tumor recurrence; thus, the potential complexities associated with late-stage tumor treatment, which may affect the reliability of the research conclusions, were excluded from consideration.

## Results

### Patients

A total of 247 male patients with liver cancer, aged 60.09 ± 13.57 years, were enrolled in this study. Among these, 208 (84.21%) were diagnosed with hepatitis B and 19 (7.69%) with hepatitis C. Additionally, 51 patients (23.17%) had diabetes. All patients had a Child-Pugh liver function score of grade A. The majority presented with single tumors, accounting for 188 cases (76.11%), whereas 59 patients (23.89%) exhibited multiple lesions. Regarding Barcelona clinic liver cancer (BCLC) staging, 205 cases (83.00%) were classified as stage A, and 42 cases (17.00%) were stage B. Notably, 138 cases (55.87%) involved tumors adjacent to other organs, and 38 cases (15.38%) involved tumors adjacent to major blood vessels, including the main portal vein and its primary and secondary branches, as well as hepatic veins and other larger vessels. All patients underwent RFA treatment and were subsequently followed up (Table 1).

**Table 1:** General patient information

Variables	Patients (*N* = 247)
Age (mean ± SD, years)	60.09 ± 13.57
Sex (male/female)	247/0 (100%/0)
Diabetes (yes/no)	51/196 (23.17%/76.83%)
Etiology (HBV/HCV/ALD/NO^a^)	208/19/14/6 (84.21%/7.69%/5.67%/2.43%)
Child-Pugh (A5/A6)	211/36 (85.43%/14.57%)
Number of lesions (single/multiple)	188/59 (76.11%/23.89%)
Lesion size (mean ± SD, mm)	2.65 ± 0.74
AFP (mean ± SD, ug/L)	332.35 ± 279.00
BCLC stage (A/B)	205/42 (83.00%/17.00%)
Adjacent to other organs^a^ (yes/no)	138/109 (55.87%/44.13%)
Adjacent blood vessel^b^ (yes/no)	38/209 (15.38%/84.62%)

a Adjacent other organs: the lesions were located around the liver and adjacent to the stomach, intestine, diaphragm, or gallbladder.

b Adjacent blood vessel: the lesions were close to the main trunk and primary and secondary branches of hepatic artery, hepatic vein, and portal vein.

### E2 and age were associated with postoperative recurrence

We employed a cubic spline regression Cox proportional hazards model to identify the factors associated with tumor recurrence following minimally invasive treatment. Regarding tumor recurrence after RFA treatment, T (*p* = 0.0026), E2 (*p* = 0.0008), tumor size (*p* = 0.0054), age (*p* = 0.0002), AFP (*p* = 0.0037), etiology (*p* = 0.0271), and adjacent to blood vessels (*p* < 0.0001) showed significant statistical significance. Among them, T (*p* = 0.0008), E2 (*p* = 0.0004), and age (*p* = 0.0001) exhibited a nonlinear correlation with tumor recurrence after minimally invasive treatment (Table 2).

**Table 2:** Results of total variable regression analysis

Factor	X^2^	*p* value
T		
Linear	14.20	0.0026^a^
Nonlinear	14.19	0.0008^a^
E2		
Linear	16.79	0.0008^a^
Nonlinear	15.83	0.0004^a^
Lesion size		
Linear	12.69	0.0054
Nonlinear	6.84	0.0327
Age		
Linear	19.98	0.0002^a^
Nonlinear	18.98	0.0001^a^
AFP		
Linear	13.46	0.0037^a^
Nonlinear	4.73	0.0941
Diabetes	0.08	0.7720
Etiology	4.88	0.0271^a^
Adjacent other organs	2.99	0.0836
BCLC stage	1.49	0.2226
Number of lesions	0.64	0.4237
Adjacent blood vessels	16.47	<0.0001^a^
Total		
Nonlinear	49.43	<0.0001^a^
Linear	98.28	<0.0001^a^

a *p* < 0.05 was considered to indicate statistical significance.

### Age was a confounding factor for E2

Given that E2 exhibits a nonlinear correlation with tumor recurrence, confounding factors are likely. To identify these confounding factors, covariates were systematically removed from the model, and a cubic spline regression Cox proportional hazard model was employed to assess the influence of E2 on the cumulative recurrence rate (CRR) after each covariate was eliminated. The analysis revealed that upon the removal of age, the *p value* for E2 shifted from statistically significant to insignificant. In contrast, the elimination of other covariates did not significantly affect the *p value* of E2 (Table 3). This finding indicates that age may serve as a confounding factor for E2.

**Table 3:** Screening and identification of confounding factors

Screening of confounding factors		
Model	*p* value of E2	E2 linearity test
All variables	0.0008	0.0004
Without age	0.0938^a^	0.0416
Without lesion size	0.0051	0.0020
Without T	0.0105	0.0045
Without AFP	0.0001	0.0001
Without Etiology	0.0010	0.0004
Without adjacent blood vessels	0.0035	0.0036
Without adjacent to other organs	<0.0001	<0.0001
Without BCLC stage	0.0009	0.0004
Without number of lesions	0.0008	0.0004
Without diabetes	0.0008	0.0003

Identification of confounding factors: E2 and age are confounding factors			
Variable		X^2^	*p* value
Independent E2	linear	5.11	0.1639
	nonlinear	3.37	0.1857
Independent Age	linear	2.84	0.4175
	nonlinear	8.66	0.0132
E2 + Age	–	–	–
E2	linear	10.22	0.0168
	nonlinear	8.14	0.0171
Age	linear	8.97	0.0297
	nonlinear	8.66	0.0132

a After removing age, the *p* value of E2 changed significantly. *p* < 0.05 was considered to indicate statistical significance.

Statistical analysis was conducted using a restricted cubic spline regression Cox proportional hazards model to further elucidate the relationship between E2 and age and their roles in tumor recurrence. This model included E2, age, and their combination as independent variables. The analysis revealed that when E2 and age were considered separately, they did not significantly impact the recurrence of liver cancer following RFA, with *p values* of 0.1639 and 0.4175, respectively (Table 3). However, when E2 and age were analyzed together in the model, they demonstrated significant statistical relevance, with *p* values of 0.0168 and 0.0297, respectively (Table 3). Consequently, E2 and age were identified as confounding factors that influence one another.

### Age influences the correlation between E2 and the recurrence of HCC following minimally invasive treatment

**Figure 1:**
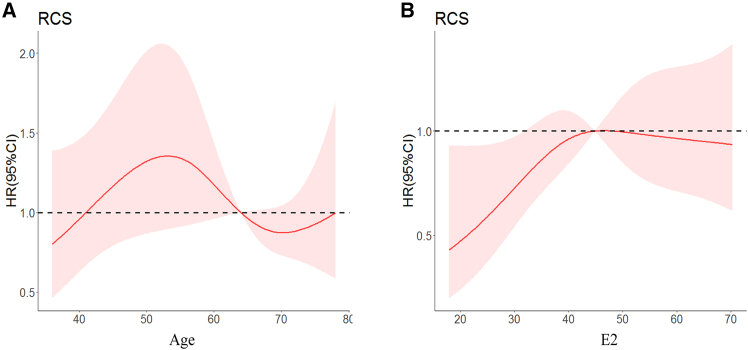
Risk trend analysis (A) The risk trend of age on the 36-month cumulative recurrence rate. (B) The risk trend of E2 on the 36-month cumulative recurrence rate.

Based on the previous findings and considering that hormone secretion is related to age, we employed the R software RMS and segmented packages to further stratify these two factors and clarify their mutual influence. The cutoff values for age were determined to be 52.75 years and 67.78 years (Figure 1A), while the cutoff value for E2 was established at 41.73 pg/mL (Figure 1B). Consequently, we categorized age into three groups: <53 years, 53–67 years, and >67 years. For E2, the categorization was as follows: <42 pg/mL and ≥42 pg/mL.

**Figure 2:**
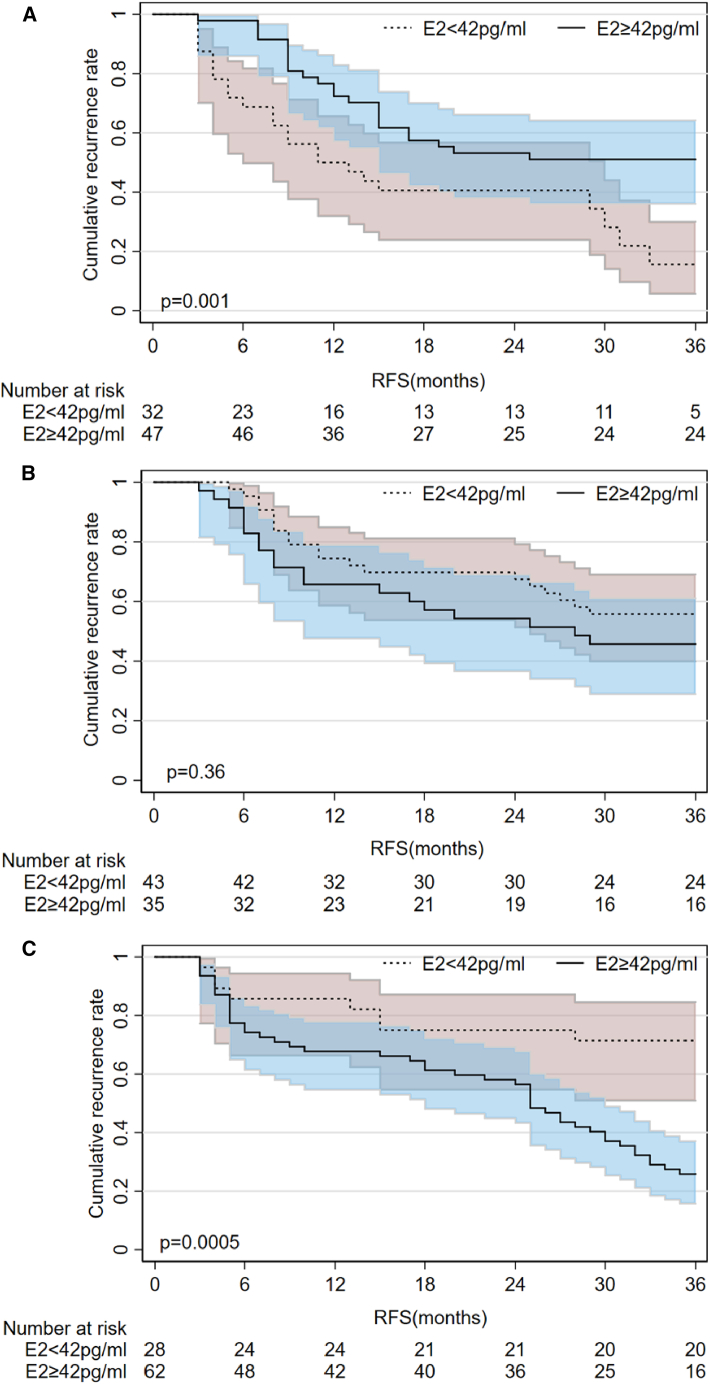
Kaplan-Meier analysis of the cumulative recurrence rate (A) Kaplan-Meier analysis of the cumulative recurrence rate for patients <53 years old. (B) Kaplan-Meier analysis of the cumulative recurrence rate for patients 53–67 years old. (C) Kaplan-Meier analysis of the cumulative recurrence rate for patients >67 years old.

We conducted the Log rank test based on the aforementioned groupings to further elucidate the impact of the two factors on patient prognosis. Our findings revealed that for patients <53 years, the median recurrence-free survival (RFS) (mRFS) was not observed in those with E2 ≥42 pg/mL. Conversely, the mRFS for patients with E2 <42 pg/mL was 12 months. Additionally, the CRR of E2 ≥42 pg/mL was significantly lower than that of E2 <42 pg/mL (*p* = 0.001, HR, 2.410; 95% CI: 1.335–4.351) (Figure 2A). When the age was 53–67 years, the mRFS of patients with E2 ≥42 pg/mL was 28 months, and the mRFS of patients with E2 <42 pg/mL was not observed. The CRR of the two groups was not statistically significant (*p* = 0.36, HR, 0.7099; 95% CI: 0.3724–1.353) (Figure 2B). When the age was >67 years, the mRFS of patients with E2 ≥42 pg/mL was 25 months, and the mRFS of patients with E2 <42 pg/mL was not observed. The CRR of patients with E2 ≥42 pg/mL was significantly higher than that of patients with E2 <42 pg/mL (*p* < 0.001, HR, 0.2935; 95% CI: 0.1690–0.5096) (Figure 2C).

Multivariate analysis was conducted using the Cox proportional hazards model, including the following variables: T, etiology, liver cirrhosis, number of lesions, chronic history of diabetes, adjacent blood vessels, AFP, lesion size, tumor BCLC stage, adjacent organs, and E2. Stepwise regression was employed for variable selection. For age <53 years, AFP, BCLC stage, and E2 were identified as significant predictors. After adjusting for AFP and BCLC stage, the CRR for E2 ≥42 pg/mL was significantly lower than that for E2 <42 pg/mL (HR: 0.409, 95% CI: 0.232–0.723, *p* = 0.002) (Table 4). For the age group of 53–67 years, E2 was retained in the model; however, only three covariates were significant: absence or chronic history of diabetes, lesion size, and proximity of the lesion to blood vessels. After adjusting for these three covariates, the CRR for E2 ≥42 pg/mL was not significantly different from that for E2 <42 pg/mL (HR: 1.934, 95% CI: 0.960–3.896, *p* = 0.065) (Table 4). For age >67 years, AFP, adjacent organs, and E2 were identified as significant variables. After adjusting for AFP and adjacent organs, the CRR for E2 ≥42 pg/mL was significantly higher than that for E2 <42 pg/mL (HR: 3.751, 95% CI: 1.752–8.029, *p* < 0.001) (Table 4).

**Table 4:** Multivariate analysis of RFS in patients of different ages

Variable	Parameter estimation	Standard error	X^2^	*p* value	HR	95% CI
Age <53 years						
AFP	0.56491	0.28766	3.8566	0.0496	1.759	1.001–3.092
E2	−0.89286	0.29026	9.4621	0.0021^a^	0.409	0.232–0.723
BCLC	1.16723	0.32468	12.9242	0.0003^a^	3.213	1.700–6.071
53≤ Age ≤67 years						
Diabetes	1.31128	0.37675	12.1142	0.0005^a^	3.711	1.773–7.765
Number of lesions	2.04131	0.40416	25.5101	<0.0001^a^	7.701	3.487–17.004
Adjacent blood vessel	1.45396	0.52871	7.5627	0.0060^a^	4.280	1.518–12.064
E2	0.65974	0.35731	3.4092	0.0648	1.934	0.960–3.896
Age >67 years						
AFP	0.75261	0.35375	4.5263	0.0334^a^	2.123	1.061–4.246
E2	1.32199	0.38833	11.5892	0.0007^a^	3.751	1.752–8.029
Adjacent other organs	−0.78809	0.29972	6.9139	0.0086^a^	0.455	0.253–0.818

a *p* < 0.05 was considered to indicate statistical significance.

## Discussion

The prospective cohort design of this study thoroughly accounts for the influence of complex real-world factors on the variables under investigation, thereby enhancing the reliability of the research findings. Firstly, this study effectively addressed the challenges posed by the variability of sex hormone levels. It is well known that sex hormone levels in the human body can be very unstable, especially periodic fluctuations in female sex hormones. This variability complicates the investigation of sex hormones. Consequently, this study focused exclusively on male patients, whose sex hormone levels tend to be more stable. Secondly, the prognosis of HCC is the key factor in this study, and the choice of various treatment options may affect it. To reduce the interference of patient outcome variables, this study only followed up on tumor recurrence, and there is only a single RFA treatment in this cohort, which further improves the reliability of the results of this study.

The impact of sex hormones on disease has long been a focal point in HCC research^3^^,^^8^^,^^19^; however, current findings in this area are conflicting.^12^ This discrepancy may be attributed to the complex mechanisms of sex hormones in the human body.^20^ We anticipate that this study will provide valuable insights into these issues.

The first important finding of this study is that age and E2 are confounding factors in the progression of HCC. Hormone expression levels are closely associated with age, particularly regarding the effects of male and female menopause^21^; however, few studies have explored this issue. Our study initially discovered a nonlinear relationship between E2 and tumor recurrence. With further analysis, it became clear that age and E2 were confounding factors. This indicates that an isolated analysis of either factor in the context of HCC progression is insufficient and may contribute to conflicting results.

Further analyses with a combination of the two factors showed that the age could be divided into three groups: <53 years, 53–67 years, and >67 years; and E2 could be divided into two stages: >42 pg/mL and <42 pg/mL. From the perspective of age segmentation, we believe that it may be related to male menopause. It is generally believed that male menopause occurs between the ages of 40 and 70 years,^22^^,^^23^ with 50–70 years being a high-risk population. A decline in T levels characterizes male menopause. Although there is a good overlap in the age range, we did not find that T levels decreased significantly with age, and E2 did not show such changes.

The analysis of the confounding factors between E2 and the HCC prognosis is the most significant and enlightening discovery in this study. When comparing patients aged <53 years to those aged >67 years, opposing roles of E2 in the HCC recurrence were observed, while for the middle group, E2 was not related to the HCC recurrence. This very interesting phenomenon has not yet been reported, leading us to speculate that E2 may reverse its effects with age, functioning as an axis centered around menopause. Menopause may serve as the pivotal point where the effects of E2 begin to reverse. The bidirectional effect of E2 may be associated with the expression of estrogen receptors ERα and ERβ. ERα is highly expressed in younger patients, where it reduces metastasis by inhibiting the HGF/IL-6 signaling pathway and downregulates VEGF^24^ and regulating the circRNA/miRNA/SMADs network.^25^ However, its methylation inactivation in older patients may promote recurrence.^26^^,^^27^ ERβ exerts a protective role by upregulating p27, arresting the cell cycle at the G1 phase,^28^ and inhibiting the pro-tumor activity of tumor-associated macrophages.^14^ Nevertheless, insufficient expression of ERβ in older patients results in a diminished protective effect.^29^ Relevant studies suggested that the formation of ERβ/ERα heterodimers may further regulate the spatiotemporal specificity of gene transcription.^30^ Current research suggests that the threshold concentration of E2 for promoting or inhibiting cancer remains unclear.^31^ This study proposed that menopause may be a primary temporal demarcation; however, substantial research is needed to further elucidate the underlying molecular mechanisms. Of course, this cohort exhibits a certain particularity in that the patients have liver cirrhosis, which weakens the liver’s capacity to inactivate estrogen. This impairment may also play a role in the reversal process previously described. However, these findings highlight that we cannot simply think that E2 uniformly promotes or inhibits the progression of liver cancer, and its mechanism may be different at different ages.

### Limitations of the study

This study had some limitations. Firstly, the sample size of enrolled patients was relatively small, and the research was a single-center study, so the generalizability of the results may be limited by factors such as region and patients’ baseline characteristics. Secondly, this research only measured two sex hormones, with no other sex hormones or indicators related to their receptors and signaling pathways included. Other hormones may also play a significant role and thus warrant investigation in future studies. In addition, the study did not conduct long-term dynamic monitoring of patients’ hormone levels, failing to capture the variation patterns of hormone levels after ablation and their temporal correlation with tumor recurrence.

## Resource availability

### Lead contact

Requests for further information and resources should be directed to and will be fulfilled by the lead contact, Xudong Gao (gao19751@163.com).

### Materials availability

This study did not generate new unique reagents, materials, or biological samples. All clinical materials and research tools used in this study are commercially available or were obtained in accordance with standard clinical research protocols and ethical approvals.

### Data and code availability

• All clinical and follow-up data reported in this study will be shared by the lead contact upon reasonable request, with strict compliance to relevant ethical guidelines and patient privacy protection regulations.
• This study used standard statistical analysis code with R and SAS software; no original custom code was generated for the research.
• Any additional information required to reanalyze the data reported in this paper is available from the lead contact upon request.

## Acknowledgments

This work is supported by the Natural Science Foundation of Beijing (grant nos 7222172 and 7232322) and the 10.13039/501100009592Beijing Municipal Science & Technology Commission no. (grant no Z161100000516222) from the Chinese Government.

## Author contributions

J.B., X.G., and S.Y. performed the statistical analysis. X.G., J.B., S.Y., Z. Zeng, Z. Zhang, and M.C. performed the study design and drafted the manuscript. N.L., W.W., K.Y., J.D., C.L., Y.Z., and J.H. performed the case studies and follow-up. J.B. and X.G. directly accessed and verified the underlying data reported in the manuscript. All co-authors contributed to the article and approved the submitted version.

## Declaration of interests

The authors have no conflicts of interest to declare.

## Declaration of generative AI and AI-assisted technologies in the writing process

No generative AI or AI-assisted technologies were used in the preparation of this manuscript. All content was independently completed and revised by the authors, who take full responsibility for the entire publication content.

## STAR★Methods

### Key resources table

**Table:** 

REAGENT or RESOURCE	SOURCE	IDENTIFIER
**Biological samples**		

Clinical human samples from study participants(peripheral blood)	Peking University Cancer Hospital & the 5th Medical Center of Chinese PLA General Hospital (Ethics No. 2016KT56)	

**Critical commercial assays**		

Roche Diagnostics	Estradiol (E2) and Testosterone (T) chemiluminescence immunoassay kit (Roche, clinical grade)	F. Hoffmann-La Roche Ltd

**Deposited data**		

The data that support the findings of this study are available from the corresponding author upon reasonable request.	Clinical, follow-up and statistical analysis data of this study	

**Software and algorithms**		

R Studio software	Posit PBC	
SAS version 9.4	SAS Institute Inc.	

### Experimental model and study participant details

This study is a prospective observational cohort study focused on human clinical participants, with **no experimental animal models, cell lines, or microbial strains** used in the research. All study procedures involving human participants were approved by the Ethics Committee of Peking University Cancer Hospital (Ethics No. 2016KT56), and written informed consent was obtained from all enrolled patients prior to study initiation, in full compliance with the ethical principles for medical research involving human subjects.

### Method details

#### Participants

This study was a prospective observational cohort study. The inclusion criteria were as follows: 1) male patients with cirrhosis; 2) age 18–80 years; 3) diagnosis of HCC in accordance with the European Association for the Study of the Liver (EASL) guidelines on the management of liver cancer and the American Association for the Study of Liver Diseases (AASLD) guidance on the diagnosis, staging, and treatment of HCC^32^^,^^33^; 4) the tumor met the indications for RFA, and the patient voluntarily agreed to undergo the procedure; and 5) the patient signed the informed consent. The exclusion criteria were as follows: 1) patients with sex hormone-related diseases or diseases affecting sex hormone levels in addition to cirrhosis, such as pituitary tumors or sexual dysfunction; 2) patients who did not receive RFA or who could not achieve complete ablation of the lesion; 3) patients participating in other research projects; 4) individuals with mental health conditions that could interfere with the study; 5) HCC patients who received Liver transplantation, surgical resection, interventional therapy, targeted therapy, or immunotherapy and so on; and 6) patients who the researcher believed were not suitable for enrollment, such as patients with potential poor compliance, etc.

This study was approved by the ethics committee of Peking University Cancer Hospital, and informed consent was obtained from all patients (Ethics No. 2016KT56). The patients or the public were not involved in the design, conduct, reporting, or dissemination plans of our research.

From October 2016 to June 2021, 263 male patients were enrolled at Peking University Cancer Hospital. Among them, one patient was excluded due to pituitary disease, three were excluded due to sexual dysfunction, four were excluded for participating in other clinical studies, five did not achieve complete ablation of the lesions, and three were excluded due to poor compliance. Ultimately, 247 patients were enrolled.

#### Protocol and indicators

Before RFA, the patients underwent routine examinations, including abdominal imaging, electrocardiography (ECG), lung computed tomography (CT), liver and kidney function tests, routine blood tests, blood coagulation time, hepatotropic virus detection, etc. In addition, we assessed the levels of the sex hormones testosterone (T) and estradiol (E2) using a Roche kit and chemiluminescence immunoassay.

We then performed an accurate assessment of the patient’s condition. The Child–Pugh score was used to evaluate liver function, while the Barcelona Clinic Liver Cancer (BCLC) staging system was employed for liver cancer staging. Tumor size was defined as the maximum cross-sectional diameter of the lesion. The term ‘adjacent to other organs' refers to tumors located at the edge of the liver, in proximity to the diaphragmatic muscle, gallbladder, stomach, intestine, and kidney. Additionally, ‘adjacent blood vessels' was defined as the closest distance between lesions and major blood vessels (i.e., hepatic vein, main hepatic artery, main portal vein, and their primary and secondary branches) being <0.5 cm.

Ultrasound-guided RFA was routinely performed (our research team took the lead in developing this technology in China in 2000 and conducted related research, resulting in a mature technical method). Following RFA, the complete ablation of lesions was confirmed through contrast-enhanced ultrasound, per the characteristics detailed in previous studies.^34^^,^^35^

Regular follow-up observations following RFA involved reviewing the patients’ abdominal imaging every 2 to 3 months. This included enhanced CT, enhanced magnetic resonance imaging (MRI), or contrast-enhanced ultrasound. In addition, monitoring of tumor markers, such as alpha-fetoprotein (AFP), was performed to assess for tumor recurrence. The follow-up period concluded upon detecting tumor recurrence or after 36 months post-RFA.

The following data were collected: demographic information, including age, gender, etc.; basic diseases, including diabetes, alcoholic liver disease, fatty liver, etc.; etiological factors, such as hepatitis B virus, hepatitis C virus, etc.; sex hormone expression level, including T and E2; tumor marker AFP; Child–Pugh score; BCLC stage; tumor size, number of lesions, adjacent to other organs, and adjacent blood vessels; and recurrence-free survival (RFS).

#### Outcomes

The main outcome was tumor recurrence after ablation treatment. This cohort study was mainly interested in the role of E2 in the recurrence of HCC after ablation. To further explore the role of E2 in the progression of liver cancer, confounding factors related to estrogen and tumor recurrence were also focused on.

### Quantification and statistical analysis

Data analysis was conducted using R Studio software and SAS version 9.4. Continuous variables were expressed as mean ± standard deviation or median (quartile) and were analyzed using either the *t* test or the Mann–Whitney U test. The relationship between E2 and the 36-month cumulative recurrence rate (CRR, CRR is defined as the percentage of patients who experienced tumor recurrence within the 36-month follow-up period, calculated as the number of cumulative recurrence events divided by the total number of enrolled patients.) was examined using the restricted cubic spline regression Cox proportional hazards model. Covariates were systematically eliminated individually to assess the confounding factors associated with E2. Based on the preliminary finding that E2 and age exhibit nonlinear correlations with HCC recurrence and act as mutual confounding factors, we employed the segmented regression method integrated with the Cox proportional hazards model to identify their optimal cutoff values. This analysis was performed using the ‘rms' and ‘segmented' packages in R software. Patients were subsequently stratified according to these cutoff points, and the significance of relevant factors in tumor progression was evaluated through survival analysis, the log rank test, and the Cox proportional hazards model.

#### Detailed statistical workflow

The specific analytical sequence was as follows (the detailed results are provided in the supplemental information).

1. The relationship between E2 and 36-month cumulative recurrence rate was analyzed by the restricted cubic spline regression Cox proportional hazards model;
2. After clarifying the relationship between E2 and the 36-month cumulative recurrence rate, the covariates were removed from the model one by one, and the impact of removing the covariates one by one on the relationship between E2 and the 36-month cumulative recurrence rate was analyzed, to analyze whether the covariate was a confounding factor of E2, and further analyze the individual effect and joint effect of E2 and the covariate;
3. After clarifying the mixed relationship between E2 and age, the age was stratified and analyzed according to different age groups based on the piecewise regression method of Cox proportional hazards model;
4. Considering that the sample size of each group is small after stratification, which may affect the stability of the restricted cubic spline regression Cox proportional hazards model, all continuous variables are stratified by the piecewise regression method of Cox proportional hazards model, and become hierarchical variables; In addition, from a clinical point of view, the results of segmented analysis are easier to understand and more easily accepted by clinicians.
5. For different age groups, with the 36-month cumulative recurrence rate as the dependent variable, E2 as the main analysis variable, and other factors as covariates, the Cox proportional hazard model was used to analyze the relationship between E2 and the 36-month cumulative recurrence rate (the grade variable was introduced into the model as a dummy variable);
6. Objective to compare the characteristics and differences of the effect of E2 on the 36-month cumulative recurrence rate in different age groups.

#### Additional resources

No additional web-based resources, study protocols, or clinical trial registries are associated with this study. All relevant research details and supporting information have been fully reported in the main manuscript and supplemental information.
