# Supplementary material for: Age modulates estradiol’s dual role in hepatocellular carcinoma recurrence after ablation: A prospective observational study
Source: iScience. 2026 Apr 21;29(6):115823. doi: 10.1016/j.isci.2026.115823 (PMC13156675; doi:10.1016/j.isci.2026.115823)
Supplement: Document S1. Figures S1–S5, Tables S1–S22, and Methods S1 [file mmc1.pdf]

iScience, Volume *29*

Supplemental information

Age modulates estradiol's dual role in hepatocellular carcinoma recurrence after ablation: A prospective observational study

Xudong Gao, Jingfeng Bi, Shuwen Yang, Zhongyi Zhang, Na Liu, Wei Wu, Kun Yan, Jinghui Dong, Changchun Liu, Yun Zhu, Jiagang Huang, Zhen Zeng, and MinHua Chen

Data S1/Methods S1: the Detailed Results for Assessing the Relationship Between E2 and the Cumulative Recurrence Rate in Hepatocellular Carcinoma After Ablation

Part I Overall analysis

1. Table S1. the baseline distribution of each independent variable. Related to Table 1

**Variable**	**Recurrence or not**	**Number**	**Median (25%, 75%)**	**Z Value**	**P Value**
Age (years)	No recurrence	105	63(45,72)	0	1
	Recurrence	142	64(47,71)		
T(ng/ml)	No recurrence	105	5.14(3.73,6.86)	0.19	0.8457
	Recurrence	142	5.1(3.72,6.36)		
E2(pg/ml)	No recurrence	105	44.12(34.23,53.58)	-1.77	0.076
	Recurrence	142	44.95(36.51,55.94)		
AFP(ng/ml)	No recurrence	105	258.05(41.79,406)	-2.42	0.0154
	Recurrence	142	359(45,582)		
Lesion size (cm)	No recurrence	105	2.4(2,3.08)	-2.24	0.025
	Recurrence	142	2.79(2.16,3.24)		

**Variable**	**Recurrence or not**	**Group**	**Frequent and continuous**	**Frequency**	**X^2**	**P Value**
Diabetes	No recurrence	Without	85	80.95	0.29	0.5932
	No recurrence	With	20	19.05	.	.
	No recurrence	Total	105	.	.	.
	Recurrence	Without	111	78.17	.	.
	Recurrence	With	31	21.83	.	.
	Recurrence	Total	142	.	.	.
Etiology	No recurrence	Without	94	89.52	3.88	0.0489
	No recurrence	With	11	10.48	.	.
	No recurrence	Total	105	.	.	.
	Recurrence	Without	114	80.28	.	.
	Recurrence	With	28	19.72	.	.
	Recurrence	Total	142	.	.	.
BCLC stage	No recurrence	A	96	91.43	9.2	0.0024
	No recurrence	B	9	8.57	.	.
	No recurrence	Total	105	.	.	.
	Recurrence	A	109	76.76	.	.
	Recurrence	B	33	23.24	.	.
	Recurrence	Total	142	.	.	.
Number of lesions	No recurrence	Single	88	83.81	5.95	0.0147
	No recurrence	Multiple	17	16.19	.	.
	No recurrence	Total	105	.	.	.
	Recurrence	Single	100	70.42	.	.
	Recurrence	Multiple	42	29.58	.	.
	Recurrence	Total	142	.	.	.

Adjacent blood vessel	No recurrence	No	96	91.43	6.51	0.0107
	No recurrence	Yes	9	8.57	.	.
	No recurrence	Total	105	.	.	.
	Recurrence	No	113	79.58	.	.
	Recurrence	Yes	29	20.42	.	.
	Recurrence	Total	142	.	.	.
Adjacent other organs	No recurrence	No	41	39.05	1.91	0.1666
	No recurrence	Yes	64	60.95	.	.
	No recurrence	Total	105	.	.	.
	Recurrence	No	68	47.89	.	.
	Recurrence	Yes	74	52.11	.	.
	Recurrence	Total	142	.	.	.

Note: With recurrence as the dependent variable, the baseline distribution of each independent variable: Diabetes: divided into without and with two groups; Etiology: history of liver disease, divided into without and with two groups; BCLC stage: divided into A and B two groups; Number of lesions, divided into single and multiple two groups; Adjacent blood vessel: whether the tumor is adjacent to blood vessels, divided into no and yes two groups; Adjacent other organs: whether it is near the liver surface, diaphragm, gallbladder, heart, digestive tract and other tissues or organs, divided into no and yes two groups.

2. Table S2 Results of total variable regression analysis. Related to Table 2.

**Factor**	**X^2**	**P Valuee**
T	14.20	0.0026
Nonlinear	14.19	0.0008
E2	16.79	0.0008
Nonlinear	15.83	0.0004
Lesion size	12.69	0.0054
Nonlinear	6.84	0.0327
Age	19.98	0.0002
Nonlinear	18.98	0.0001
AFP	13.46	0.0037
Nonlinear	4.73	0.0941
Diabetes	0.08	0.7720
Etiology	4.88	0.0271
Adjacent other organs	2.99	0.0836
BCLC stage	1.49	0.2226
Number of lesions	0.64	0.4237
Adjacent blood vessel	16.47	<.0001
Total Nonlinear	49.43	<.0001
Total	98.28	<.0001

3. Table S3. Regression analysis results after removing Age

**Factor**	**Chi-Square**	**d.f.**	**P**

T	7.84	3	0.0494
Nonlinear	7.83	2	0.0199
E2	6.4	3	0.0938
Nonlinear	6.36	2	0.0416
Lesion size	10.78	3	0.013
Nonlinear	5.69	2	0.058
AFP	20.9	3	0.0001
Nonlinear	11.13	2	0.0038
Diabetes	0.01	1	0.9071
Etiology	1.84	1	0.1749
Adjacent other organs	5.27	1	0.0217
BCLC stage	2.24	1	0.1341
Number of lesions	0.25	1	0.6142
Adjacent blood vessel	11.73	1	0.0006
Total Nonlinear	28.09	8	0.0005
Total	77.64	19	<.0001

4.Table S4. Regression analysis results after removing Lesion size.

**Factor**	**Chi-Square**	**d.f.**	**P**
T	13.72	3	0.0033
Nonlinear	13.69	2	0.0011
E2	12.8	3	0.0051
Nonlinear	12.47	2	0.0020
Age	17.2	3	0.0006
Nonlinear	17.09	2	0.0002
AFP	13.66	3	0.0034
Nonlinear	5.02	2	0.0815
Diabetes	0.4	1	0.5281
Etiology	5.39	1	0.0202
Adjacent other organs	4.41	1	0.0357
BCLC stage	1.92	1	0.1662
Number of lesions	0.74	1	0.3893
Adjacent blood vessel	13.83	1	0.0002
Total Nonlinear	40.39	8	<.0001
Total	85.75	19	<.0001

5. Table S5. Regression analysis results after removing T.

**Factor**	**Chi-Square**	**d.f.**	**P**
E2	11.23	3	0.0105
Nonlinear	10.80	2	0.0045
Lesion size	12.35	3	0.0063
Nonlinear	6.33	2	0.0422
Age	14.21	3	0.0026

Nonlinear	13.71	2	0.0011
AFP	13.64	3	0.0034
Nonlinear	5.63	2	0.0598
Diabetes	0.07	1	0.7899
Etiology	1.16	1	0.2819
Adjacent other organs	3.72	1	0.0537
BCLC stage	1.79	1	0.1815
Number of lesions	1.89	1	0.1692
Adjacent blood vessel	15.52	1	0.0001
Total Nonlinear	36.83	8	<.0001
Total	87.24	19	<.0001

6. Table S6. Regression analysis results after removing AFP.

**Factor**	**Chi-Square**	**d.f.**	**P**
T	14.8	3	0.0020
Nonlinear	14.8	2	0.0006
E2	21.55	3	0.0001
Nonlinear	17.85	2	0.0001
Lesion size	12.65	3	0.0055
Nonlinear	6.24	2	0.0441
Age	27.81	3	<.0001
Nonlinear	27.57	2	<.0001
Diabetes	0.00	1	0.9544
Etiology	6.99	1	0.0082
Adjacent other organs	2.99	1	0.0836
BCLC Stage	1.69	1	0.1942
Number of lesions	1.11	1	0.2926
Adjacent blood vessel	15.4	1	0.0001
Total Nonlinear	44.85	8	<.0001
Total	82.77	19	<.0001

7.Table S7. Regression analysis results after removing Etiology.

**Factor**	**Chi-Square**	**d.f.**	**P**
T	10.97	3	0.0119
Nonlinear	10.94	2	0.0042
E2	16.2	3	0.001
Nonlinear	15.81	2	0.0004
Lesion size	13.1	3	0.0044
Nonlinear	7.34	2	0.0255
Age	17.45	3	0.0006
Nonlinear	16.34	2	0.0003
AFP	15.47	3	0.0015
Nonlinear	6.45	2	0.0397

Diabetes	0.32	1	0.5707
Adjacent other organs	1.22	1	0.2692
BCLC Stage	5.29	1	0.0215
Number of lesions	0.14	1	0.7034
Adjacent blood vessel	14.92	1	0.0001
Total Nonlinear	46.92	10	<.0001
Total	95.99	21	<.0001

8. Table S8. Regression analysis results after excluding Adjacent blood vessel.

**Factor**	**Chi-Square**	**d.f.**	**P**
T	12.4	3	0.0061
Nonlinear	11.38	2	0.0034
E2	13.59	3	0.0035
Nonlinear	11.23	2	0.0036
Lesion size	10.45	3	0.0151
Nonlinear	6.18	2	0.0454
Age	16.01	3	0.0011
Nonlinear	14.3	2	0.0008
AFP	12.38	3	0.0062
Nonlinear	4.02	2	0.134
Diabetes	0.27	1	0.6063
Adjacent other organs	5.77	1	0.0163
Etiology	3.5	1	0.0612
BCLC stage	2.99	1	0.0836
Number of lesions	0.03	1	0.8529
Total Nonlinear	40.48	10	<.0001
Total	87.3	21	<.0001

9. Table S9. Regression analysis results after excluding Adjacent other organs.

**Factor**	**Chi-Square**	**d.f.**	**P**
T	14.81	3	0.002
Nonlinear	14.81	2	0.0006
E2	22.59	3	<.0001
Nonlinear	22.42	2	<.0001
Lesion size	13.99	3	0.0029
Nonlinear	7.63	2	0.022
Age	21.92	3	0.0001
Nonlinear	19.01	2	0.0001
AFP	13.31	3	0.004
Nonlinear	4.75	2	0.0928
Diabetes	0.16	1	0.6891
Etiology	3.15	1	0.0758
BCLC stage	2.77	1	0.0963

Number of lesions	0.2	1	0.6569
Adjacent blood vessel	19.88	1	<.0001
Total Nonlinear	54.72	10	<.0001
Total	93.82	21	<.0001

10.Table S10. Regression analysis results after excluding BCLC stage.

**Factor**	**Chi-Square**	**d.f.**	**P**
T	15.05	3	0.0018
Nonlinear	15.04	2	0.0005
E2	16.55	3	0.0009
Nonlinear	15.52	2	0.0004
Lesion size	13.13	3	0.0044
Nonlinear	6.71	2	0.0349
Age	20.85	3	0.0001
Nonlinear	20.04	2	<.0001
AFP	13.81	3	0.0032
Nonlinear	4.56	2	0.1020
Diabetes	0.15	1	0.6979
Etiology	9.25	1	0.0024
Adjacent other organs	4.41	1	0.0358
Number of lesions	8.47	1	0.0036
Adjacent blood vessel	18.36	1	<.0001
Total Nonlinear	50.31	10	<.0001
Total	96.41	21	<.0001

11.Table S11. Regression analysis results after excluding Number of lesions.

**Factor**	**Chi-Square**	**d.f.**	**P**
T	14.61	3	0.0022
Nonlinear	14.6	2	0.0007
E2	16.71	3	0.0008
Nonlinear	15.68	2	0.0004
Lesion size	12.79	3	0.0051
Nonlinear	6.91	2	0.0316
Age	19.52	3	0.0002
Nonlinear	18.34	2	0.0001
AFP	13.8	3	0.0032
Nonlinear	5.03	2	0.0808
Diabetes	0.06	1	0.8059
Etiology	4.44	1	0.0351
Adjacent other organs	2.58	1	0.108
BCLC stage	9.6	1	0.0019
Adjacent blood vessel	15.88	1	0.0001
Total Nonlinear	49.34	10	<.0001

Total	98.02	21	<.0001

12. Table S12. Regression analysis results after excluding Diabetes.

Factor	Chi-Square	d.f.	P
T	14.13	3	0.0027
Nonlinear	14.12	2	0.0009
E2	16.79	3	0.0008
Nonlinear	16.07	2	0.0003
Lesion size	13.03	3	0.0046
Nonlinear	6.91	2	0.0315
Age	19.87	3	0.0002
Nonlinear	19.01	2	0.0001
AFP	13.34	3	0.0039
Nonlinear	4.67	2	0.0970
Etiology	5.13	1	0.0235
Adjacent other organs	3.07	1	0.0799
BCLC stage	1.55	1	0.2131
Adjacent blood vessel	16.67	1	<.0001
Number of lesions	0.62	1	0.4328
Total Nonlinear	49.8	10	<.0001
Total	98.15	21	<.0001

13. Table S13. Summary of results. Related to Table 3

Model	P Value of E2	E2 Linearity Test
All variables	0.0008	0.0004
Other variables except Age	0.0938	0.0416
Other variables except Lesion size	0.0051	0.0020
Other variables except T	0.0105	0.0045
Other variables except AFP	0.0001	0.0001
Other variables except Etiology	0.0010	0.0004
Other variables except Adjacent blood vessel	0.0035	0.0036
Other variables except Adjacent other organs	<0.0001	<0.0001
Other variables except BCLC stage	0.0009	0.0004
Other variables except Number of lesions	0.0008	0.0004
Other variables except Diabetes	0.0008	0.0003

Note: Table S13 shows that when Age is removed, the P value of E2 changes from statistically significant to non statistically significant, suggesting that Age has a greater impact on E2.

14. Table S14 Interaction between E2 and Age (Related to Table 3)

**Variable**	X^2	**P Value**
**Independent** E2	5.11	0.1639
Nonlinear	3.37	0.1857
**Independent** Age	2.84	0.4175

Nonlinear	8.66	0.0132
E2 + Age:		
E2	10.22	0.0168
Nonlinear	8.14	0.0171
Age	8.97	0.0297
Nonlinear	8.66	0.0132

15. Table S15. Results of piecewise regression.

Variable	R Software results		Actual segmentation		
Age	52.75	67.78	<53	53-67	>67
E2	41.73	-	<42	≥42	-
T	3.726	6.231	<3.73	3.73-6.23	>6.23
Lesion size	2.175	3.152	<2.18	2.18-3.15	>3.15
AFP	217.7	626.8	<218	218-627	>627

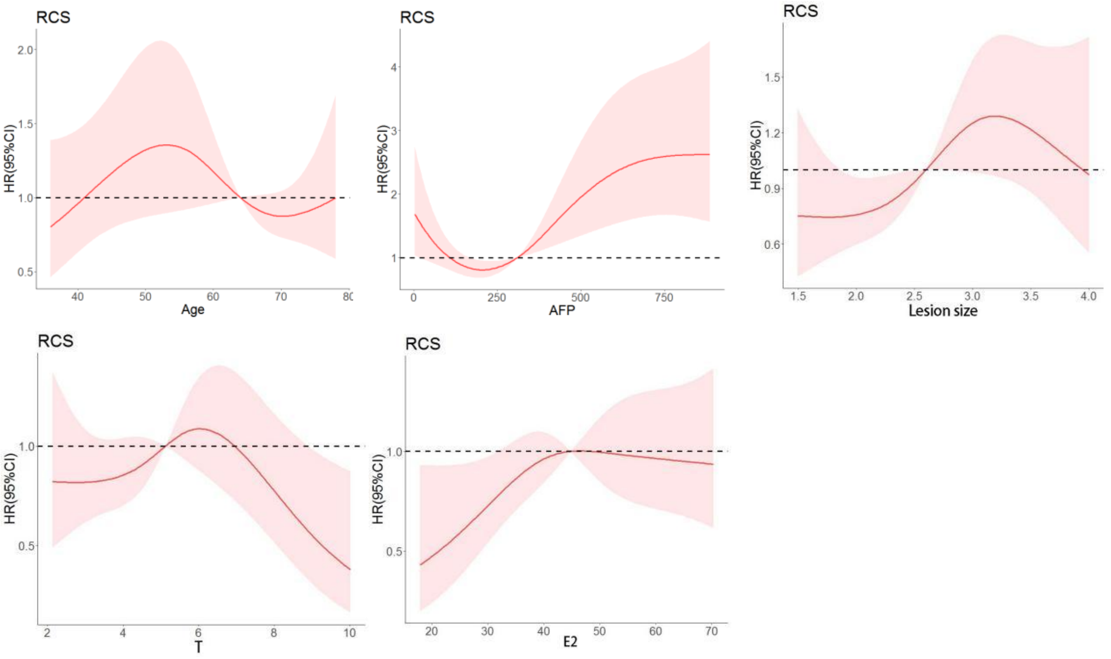

Figure S1 Risk trend of Age, E2, T, Lesion size and AFP on 36-month cumulative recurrence rate. Related to Figure 1.

Part II Analysis of Age<53 years old group

1. Table S16. Baseline of indicators between E2 and control group

Variable	E2	Group	Frequent and continuous	Frequency	X^2	P Value
T	<42	<3.73	17	53.13	11.7	0.0029
	<42	3.73-6.23	10	31.25	.	.
	<42	>6.23	5	15.63	.	.
	<42	total	32	.	.	.
	≥42	<3.73	8	17.02	.	.
	≥42	3.73-6.23	23	48.94	.	.

	≥42	>6.23	16	34.04	.	.
	≥42	total	47	.	.	.
Diabetes	<42	Without	32	100	.	0.5118
	<42	With	0	0	.	.
	<42	total	32	.	.	.
	≥42	Without	45	95.74	.	.
	≥42	With	2	4.26	.	.
	≥42	total	47	.	.	.
Etiology	<42	Without	21	65.63	.	0
	<42	With	11	34.38	.	.
	<42	total	32	.	.	.
	≥42	Without	47	100	.	.
	≥42	With	0	0	.	.
	≥42	total	47	.	.	.
BCLC stage	<42	A	21	65.63	3.14	0.0765
	<42	B	11	34.38	.	.
	<42	total	32	.	.	.
	≥42	A	39	82.98	.	.
	≥42	B	8	17.02	.	.
	≥42	total	47	.	.	.
Number of Lesions	<42	Single	21	65.63	1.14	0.2856
	<42	Multiple	11	34.38	.	.
	<42	total	32	.	.	.
	≥42	Single	36	76.6	.	.
	≥42	Multiple	11	23.4	.	.
	≥42	total	47	.	.	.
Adjacent blood vessel	<42	No	32	100	15.87	0.0001
	<42	Yes	0	0	.	.
	<42	total	32	.	.	.
	≥42	No	29	61.7	.	.
	≥42	Yes	18	38.3	.	.
	≥42	total	47	.	.	.
AFP	<42	<218	13	40.63	.	0.2676
	<42	218-627	16	50	.	.
	<42	>627	3	9.38	.	.
	<42	total	32	.	.	.
	≥42	<218	25	53.19	.	.
	≥42	218-627	15	31.91	.	.
	≥42	>627	7	14.89	.	.
	≥42	total	47	.	.	.
Lesion Size	<42	<2.18	6	18.75	.	0
	<42	2.18-3.15	25	78.13	.	.
	<42	>3.15	1	3.13	.	.
	<42	total	32	.	.	.

	≥42	<2.18	37	78.72	.	.
	≥42	2.18-3.15	10	21.28	.	.
	≥42	>3.15	0	0	.	.
	≥42	total	47	.	.	.
Adjacent other organs	<42	No	24	75	0.03	0.8706
	<42	Yes	8	25	.	.
	<42	total	32	.	.	.
	≥42	No	36	76.6	.	.
	≥42	Yes	11	23.4	.	.
	≥42	total	47	.	.	.

Note: The 36-month cumulative recurrence rate was used as the dependent variable, and the Log-rank test was performed for each variable. There was only one case with Lesion size=3, which was combined with Lesion size=2; Diabetes1=1 only 2 cases were not analyzed.

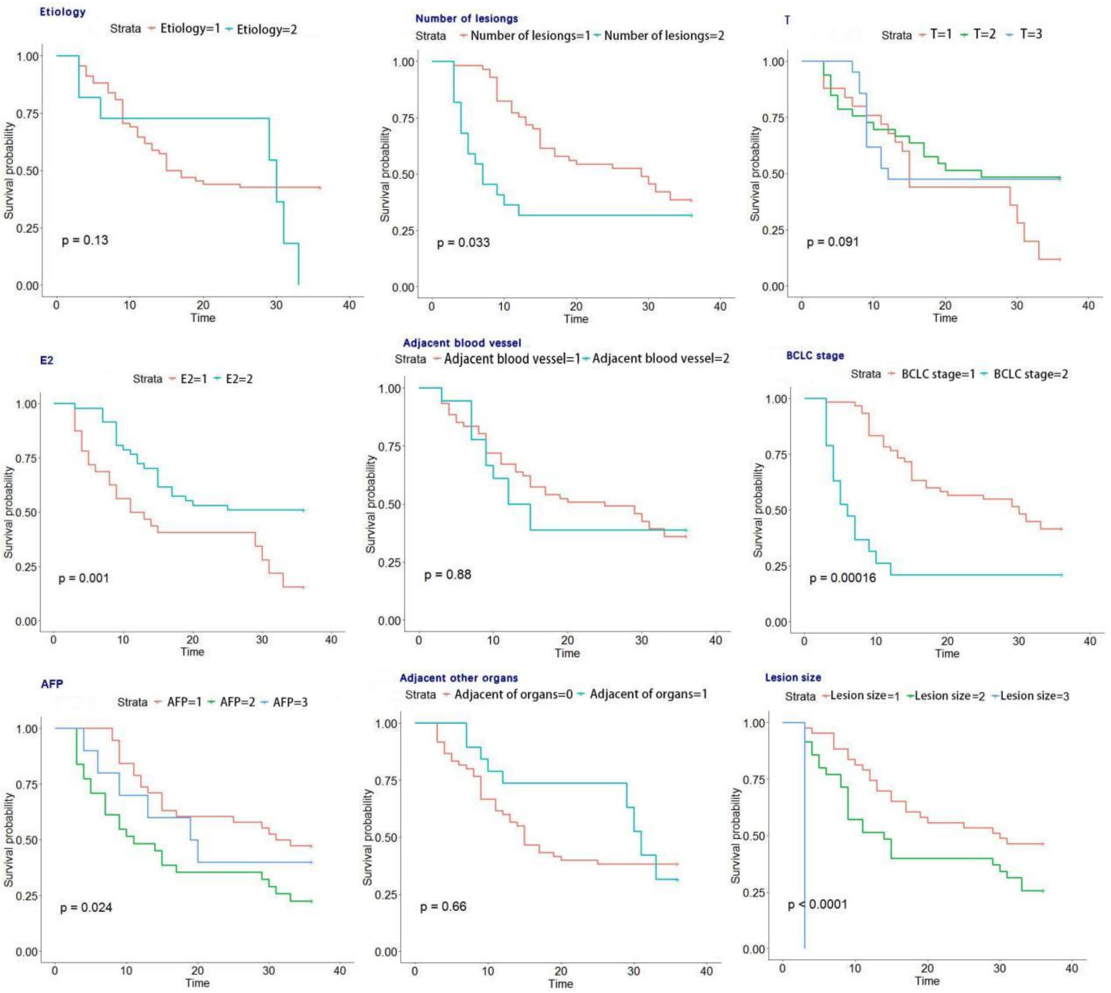

Fig. S2 Age<53, with 36 month cumulative recurrence rate as the dependent variable, the Log-rank test results of each variable. Related to Figure 2.

2.Table S17 Stepwise regression results of all variables. Related to Table 4.

Maximum likelihood estimation analysis								
**Parameter**	**Freedom**	**Parameter estimation**	**Standard Error**	**X^2**	**P Value**	**HR**	**95% CI**	
**AFPgroup1**	1	0.69182	0.30784	5.0506	0.0246	1.997	1.093	3.652
**AFPgroup2**	1	0.71125	0.48342	2.1647	0.1412	2.037	0.790	5.253
**E2group**	1	-0.96334	0.29693	10.5258	0.0012	0.382	0.213	0.683
**BCLC stage**	1	1.22671	0.33112	13.7252	0.0002	3.410	1.782	6.525

Note: taking the 36-month cumulative recurrence rate as the dependent variable (recurrence=1, no recurrence=0),T (with<3.73 as the reference group), Etiology (with liver disease history=1, no liver disease history=0),Number of Lesions (single lesion=1, multiple lesions=2), Diabetes (without diabetes =0, with diabetes =1), Adjacent blood vessel (adjacent vessels=1, no adjacent vessels=0), AFP (with<218 as the reference group), Lesion Size (with<2.18 as the reference

group), BCLC stage (A=1, B=2)，Adjacent other organs (adjacent to other organs or tissues=1, not adjacent=0) and E2 (increase=2, decrease=1) were independent variables.

Part III Analysis of 53 ≤ Age ≤ 67 years old group

1. Table S18. E2 baseline of each indicator between the two groups

Variable	E2	Group	Frequent and continuous	Frequency	X^2	P Value
T	<42	<3.73	17	39.53	8.06	0.0178
	<42	3.73-6.23	17	39.53	.	.
	<42	>6.23	9	20.93	.	.
	<42	total	43	.	.	.
	≥42	<3.73	5	14.29	.	.
	≥42	3.73-6.23	14	40	.	.
	≥42	>6.23	16	45.71	.	.
	≥42	total	35	.	.	.
Diabetes	<42	Without	28	65.12	2.11	0.1463
	<42	With	15	34.88	.	.
	<42	total	43	.	.	.
	≥42	Without	28	80	.	.
	≥42	With	7	20	.	.
	≥42	total	35	.	.	.
Etiology	<42	Without	38	88.37	.	0.061
	<42	With	5	11.63	.	.
	<42	total	43	.	.	.
	≥42	Without	35	100	.	.
	≥42	With	0	0	.	.
	≥42	total	35	.	.	.
BCLC stage	<42	A	38	88.37	.	0.4497
	<42	B	5	11.63	.	.
	<42	total	43	.	.	.
	≥42	A	33	94.29	.	.
	≥42	B	2	5.71	.	.
	≥42	total	35	.	.	.
Number of lesions	<42	Single	38	88.37	1.75	0.1857
	<42	Multiple	5	11.63	.	.
	<42	total	43	.	.	.
	≥42	Single	27	77.14	.	.
	≥42	Multiple	8	22.86	.	.
	≥42	total	35	.	.	.

Adjacent blood vessel	<42	No	37	86.05	.	0.1219
	<42	Yes	6	13.95	.	.
	<42	total	43	.	.	.
	≥42	No	34	97.14	.	.
	≥42	Yes	1	2.86	.	.
	≥42	total	35	.	.	.
AFP	<42	<218	21	48.84	5.64	0.0595
	<42	218-627	17	39.53	.	.
	<42	>627	5	11.63	.	.
	<42	total	43	.	.	.
	≥42	<218	8	22.86	.	.
	≥42	218-627	20	57.14	.	.
	≥42	>627	7	20	.	.
	≥42	total	35	.	.	.
Lesion size	<42	<2.18	7	16.28	1.24	0.5386
	<42	2.18-3.15	16	37.21	.	.
	<42	>3.15	20	46.51	.	.
	<42	total	43	.	.	.
	≥42	<2.18	9	25.71	.	.
	≥42	2.18-3.15	13	37.14	.	.
	≥42	>3.15	13	37.14	.	.
	≥42	total	35	.	.	.
Adjacent other organs	<42	No	8	18.6	9.35	0.0022
	<42	Yes	35	81.4	.	.
	<42	total	43	.	.	.
	≥42	No	18	51.43	.	.
	≥42	Yes	17	48.57	.	.
	≥42	total	35	.	.	.

Note: The 36-month cumulative recurrence rate was used as the dependent variable, and the Log-rank test was performed for each variable.

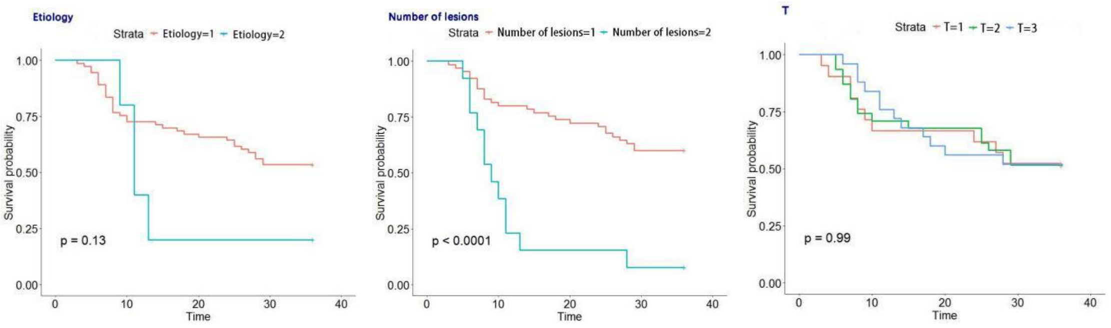

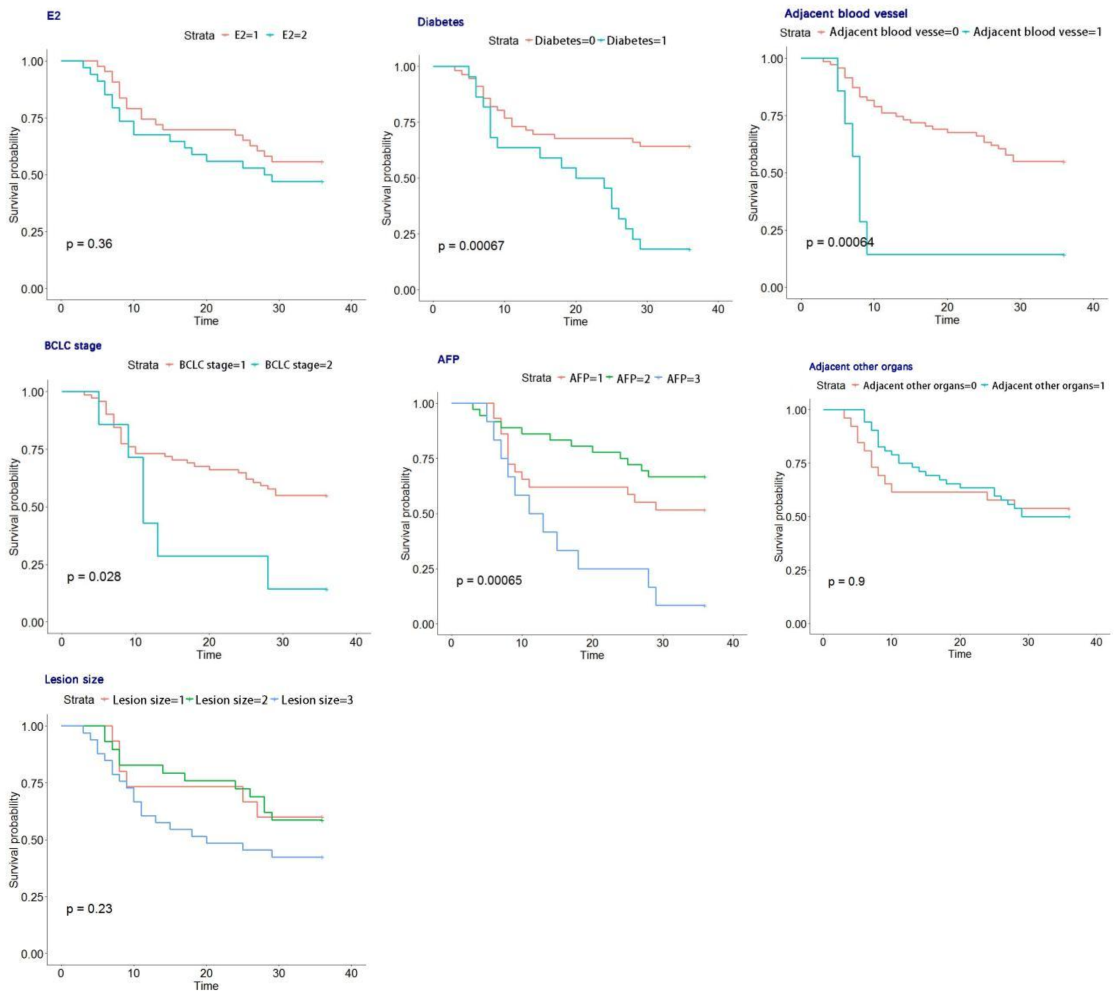

Fig. S3 Log-rank test results of 36-month cumulative recurrence rate as dependent variable in the age group of 53 ≤ age ≤ 67. Related to Figure 2.

2. Table S19 Stepwise regression results of all variables. Related to Table 4.

Maximum likelihood estimation analysis

Parameter	Freedom	Parameter Estimate	Standard Error	X^2	P Value	HR	95% CI	
Diabetes	1	1.26426	0.38086	11.0190	0.0009	3.540	1.678	7.469
Number of lesions	1	2.11843	0.40785	26.9786	<.0001	8.318	3.740	18.501
Adjacent blood vessel	1	1.21419	0.50309	5.8250	0.0158	3.368	1.256	9.027

Note: taking the 36-month cumulative recurrence rate as the dependent variable (recurrence=1, no recurrence=0),T (with<3.73 as the reference group), Etiology (with liver disease history=1, no liver disease history=0),Number of Lesions (single lesion=1, multiple lesions=2), Diabetes (without diabetes =0, with diabetes =1), Adjacent blood vessel (adjacent vessels=1, no adjacent vessels=0), AFP (with<218 as the reference group), Lesion Size (with<2.18 as the control group), BCLC stage (A=1, B=2)，Adjacent other organs (adjacent to other organs or tissues=1, not adjacent=0) and E2 (increase=2, decrease=1) were independent variables.

3. Table S20 All variable regression+E2 regression results. Related to Table 4.

Maximum likelihood estimation analysis								
Parameter	Freedom	Parameter Estimate	Standard Error	X^2	P Value	HR	95% CI	
Diabetes	1	1.31128	0.37675	12.1142	0.0005	3.711	1.773	7.765
Number of Lesions	1	2.04131	0.40416	25.5101	<.0001	7.701	3.487	17.004
Adjacent blood vessel	1	1.45396	0.52871	7.5627	0.0060	4.280	1.518	12.064
E2group	1	0.65974	0.35731	3.4092	0.0648	1.934	0.960	3.896

Part IV Analysis of Age>67 age group

1. Table S21 baseline of indicators between E2group

Variable	E2	Group	Frequent and continuous	Frequency	X^2	P Value
T group	<42	<3.73	15	53.57	.	0
	<42	3.73-6.23	3	10.71	.	.
	<42	>6.23	10	35.71	.	.
	<42	total	28	.	.	.
	≥42	<3.73	0	0	.	.
	≥42	3.73-6.23	50	80.65	.	.
	≥42	>6.23	12	19.35	.	.
	≥42	total	62	.	.	.
Diabetes	<42	Without	15	53.57	5.22	0.0223
	<42	With	13	46.43	.	.
	<42	total	28	.	.	.
	≥42	Without	48	77.42	.	.
	≥42	With	14	22.58	.	.
	≥42	total	62	.	.	.
Etiology	<42	Without	23	82.14	1.27	0.2605
	<42	With	5	17.86	.	.
	<42	total	28	.	.	.
	≥42	Without	44	70.97	.	.
	≥42	With	18	29.03	.	.
	≥42	total	62	.	.	.
BCLC stage	<42	A	28	100	.	0.002
	<42	B	0	0	.	.
	<42	total	28	.	.	.
	≥42	A	46	74.19	.	.
	≥42	B	16	25.81	.	.
	≥42	total	62	.	.	.
Number of lesions	<42	Single	28	100	14.78	0.0001
	<42	Multiple	0	0	.	.

	<42	total	28	.	.	.
	≥42	Single	38	61.29	.	.
	≥42	Multiple	24	38.71	.	.
	≥42	total	62	.	.	.
Adjacent blood vessel	<42	No	27	96.43	.	0.0568
	<42	Yes	1	3.57	.	.
	<42	total	28	.	.	.
	≥42	No	50	80.65	.	.
	≥42	Yes	12	19.35	.	.
	≥42	total	62	.	.	.
AFP	<42	<218	12	42.86	.	0.6208
	<42	218-627	12	42.86	.	.
	<42	>627	4	14.29	.	.
	<42	total	28	.	.	.
	≥42	<218	19	30.65	.	.
	≥42	218-627	31	50	.	.
	≥42	>627	12	19.35	.	.
	≥42	total	62	.	.	.
Lesion size	<42	<2.18	13	46.43	22.17	0
	<42	2.18-3.15	13	46.43	.	.
	<42	>3.15	2	7.14	.	.
	<42	total	28	.	.	.
	≥42	<2.18	6	9.68	.	.
	≥42	2.18-3.15	25	40.32	.	.
	≥42	>3.15	31	50	.	.
	≥42	total	62	.	.	.
Adjacent other organs	<42	No	10	35.71	2.2	0.1376
	<42	Yes	18	64.29	.	.
	<42	total	28	.	.	.
	≥42	No	13	20.97	.	.
	≥42	Yes	49	79.03	.	.
	≥42	total	62	.	.	.

Note: The 36-month cumulative recurrence rate was used as the dependent variable, and the Log-rank test was performed for each variable.

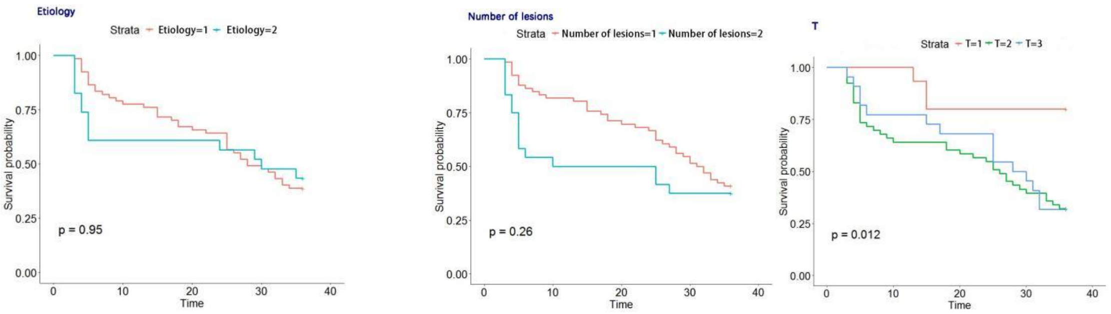

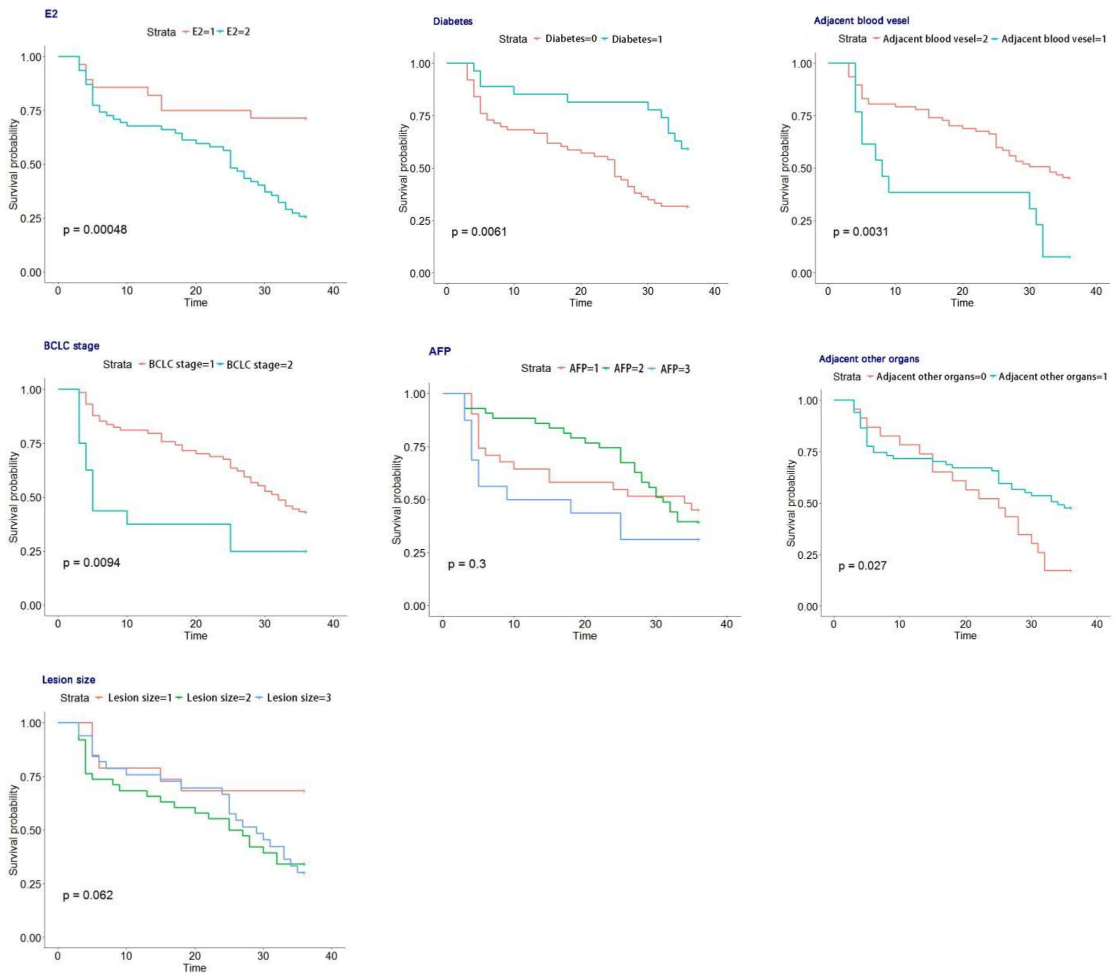

Fig. S4 Log-rank test results of 36-month cumulative recurrence rate as dependent variable in Age>67 age group. Related to Figure 2.

2. Table S22 Stepwise regression results of all variables. Related to Table 4.

Maximum likelihood estimation analysis

Parameter	Freedom	Parameter estimate	Standard error	X^2	P Value	HR	95% CI	
**AFP**	1	0.75261	0.35375	4.5263	0.0334	2.123	1.061	4.246
**E2**	1	1.32199	0.38833	11.5892	0.0007	3.751	1.752	8.029
**Adjacent**	1	-0.78809	0.29972	6.9139	0.0086	0.455	0.253	0.818

Note: the cumulative recurrence rate at 36 months was taken as the dependent variable (recurrence=1, no recurrence=0),T group (with<3.73 as the reference group), Etiology (with liver disease history=1, no liver disease history=0),Number of Lesions (single lesion=1, multiple lesions=2), Diabetes (without Diabetes =0, with Diabetes =1), Adjacent blood vessel (adjacent vessels=1, no adjacent vessels=0), AFP (with<218 as the reference group), Lesion Size group (with<2.18 as the control group), BCLC stage (A=1, B=2)，Adjacent other organs (adjacent to other organs or tissues=1, not adjacent=0) and E2 group (increase=2, decrease=1) were independent variables.

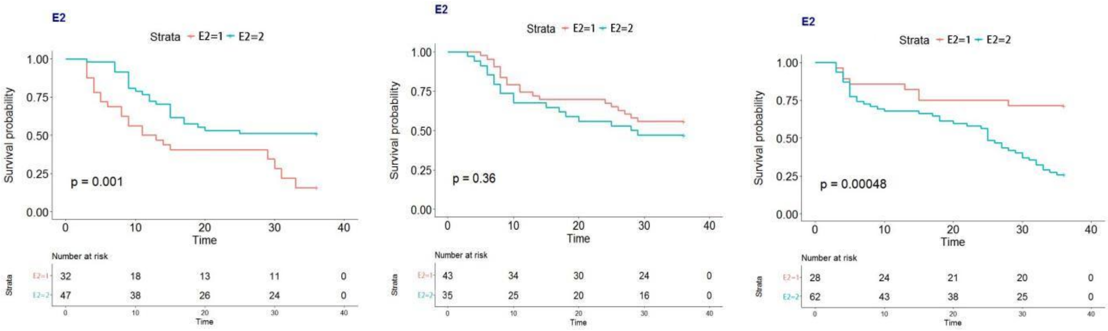

Age<53 Age 53-67 Age>67

Fig. S5 Log-rank test results of E2 at different ages. Related to Figure 2.
